# LED Arrays as Cost Effective and Efficient Light Sources for Widefield Microscopy

**DOI:** 10.1371/journal.pone.0002146

**Published:** 2008-05-14

**Authors:** Dinu F. Albeanu, Edward Soucy, Tomokazu F. Sato, Markus Meister, Venkatesh N. Murthy

**Affiliations:** 1 Program in Neuroscience, Harvard Medical School, Boston, Massachusetts, United States of America; 2 Center for Brain Science, Harvard University, Cambridge, Massachusetts, United States of America; 3 Harvard Biophysics Program, Harvard Medical School, Boston, Massachusetts, United States of America; 4 Department of Molecular and Cellular Biology, Harvard University, Cambridge, Massachusetts, United States of America; University of Washington, United States of America

## Abstract

New developments in fluorophores as well as in detection methods have fueled the rapid growth of optical imaging in the life sciences. Commercial widefield microscopes generally use arc lamps, excitation/emission filters and shutters for fluorescence imaging. These components can be expensive, difficult to maintain and preclude stable illumination. Here, we describe methods to construct inexpensive and easy-to-use light sources for optical microscopy using light-emitting diodes (LEDs). We also provide examples of its applicability to biological fluorescence imaging.

## Introduction

Advances in technology have made imaging increasingly popular in the life sciences [1]–[4]. Optical imaging permits simultaneous monitoring of large populations of cells at high spatial resolution and selective visualization of components of cells and tissues. In addition, recent progress in the design and implementation of biological fluorescence probes promises even greater expansion of fluorescence imaging [1]–[4]. For widefield imaging, traditional lighting methods have used xenon, mercury, or halogen lamps combined with excitation and emission filters in microscopes, as well as shuttering devices to control illumination duration. The cost of these parts can be significant, especially when stable and fast multi-color imaging is needed. This is because stable illumination requires a highly-regulated voltage supply, and multicolor imaging requires shutters, filter wheels or polychromators. Also, arc lamps are inherently unstable due to fast changes in the electron density in the generated plasma (plasma oscillations) and thermal runaway. In addition, irises and neutral density filters are required to regulate the light intensity. Light emitting diodes (LEDs) offer a cheap, stable and easy-to-use alternative to the traditional lighting sources. The advantages of LEDs over conventional wide-field illuminators such as arc lamps have been discussed in detail elsewhere [5], and can also be found on the World Wide Web (for example, http://www.olympusfluoview.com/theory/noncoherentsources.html).

The phenomenon of electroluminescence was discovered 100 years ago by Henry Round [6]. It was independently discovered by Oleg Losev, who also went on to develop LEDs (see reference [7] for a commentary). The commercial and scientific use of LEDs has seen exponential growth in recent years. There are two main types of LED chips: edge emitters and surface emitters. Surface emitters usually have a Lambertian emission pattern, with the intensity proportional to the cosine of the emission angle, measured from the axis perpendicular to the surface. Edge emitters typically emit from a small spot (or slit) of about 50 µm, have more complex emission pattern and potentially higher output flux than surface emitters. However, ordinary edge-emitting LEDs usually emit from all four sides of the square die in which they are set. To focus and collimate the light from all four sides, the die is placed inside a reflective cup, but this ends up expanding the effective source area considerably, making the coupling to a fiber more inefficient (see below).

LED chips are widely available now – some commercial vendors include Cree, Inc. http://www.cree.com/ (Durham, North Carolina), Epitex, Inc. http://www.epitex.com/new_global/led.html (Kyoto, Japan), Lamina Ceramics Inc, http://laminaceramics.com (Westhampton, New Jersey), Lumex, Inc. http://www.lumex.com/ (Palatine, Illinois), Luminus Devices http://www.luminus.com (Billerica, Massachusetts), Marubeni, Inc. http://tech-led.com/index.shtml (Santa Clara, California), Osram GmbH, http://www.osram.com/ (Munich, Germany), Phillips Lumileds Lighting Company http://www.lumileds.com/ (San Jose, California), Roithner Lasertechnik GmbH http://www.roithner-laser.com/ (Vienna, Austria) or Seoul Semiconductor Inc., http://www.zled.com (Seoul, Korea).

The optical output of an LED (for example, the total radiant flux) is approximately proportional to the drive current. The design and choice of the power supply used to drive the LED must take into account the response time of LEDs (which is on the order of a microsecond), the nonlinearity in the voltage-emisison relation and the maximum recommended driving current. Additional consideration should also include the intrinsic noise levels of LEDs. Salzberg et al. [8] found that the fractional fluctuation at a given intensity was less than 2×10^−5^ (over a bandwidth of 1 KHz), an order of magnitude smaller than that of a quartz tungsten halogen lamp.

Some of the commercially available LEDs mentioned above have sufficient light intensities for use in biological imaging (for example, emission centered on 470 nm or 530 nm). Since many fluorescent probes currently used in biology have relatively wide excitation spectra, LEDs of a few wavelengths should be sufficient for the excitation of most fluorophores. LEDs can be turned on and off rapidly (<1 µs), therefore the need for shuttering (∼10 ms time constant) is obviated. Although the relation between input current and light output can be nonlinear, it can be calibrated precisely. Alternately, more linear control can be achieved using pulse-width modulation. One can linearly modulate the effective LED intensity by varying the time that a flickering LED spends in the on state relative to the off state, using a digital control line. This feature allows modulating the intensity of light in a relatively repeatable manner by altering the driving current, therefore dispensing with neutral density filters. Other advantages of LEDs over arc lamps include their price (10–100 times lower), long lifetimes (∼50,000 hrs vs. <1000 hrs), their relatively harmless failure mode and the use of low voltage direct current for driving them (especially important for reducing noise in electrophysiology experiments).

Microscopes generally use arc lamps combined with excitation and emission filters because of the wide range of output wavelengths. Mechanical shutters are also used because of slow response times of the light source. Additionally, in experiments involving light of multiple wavelengths, either manual or motorized switching of filters has been used. The cost and complexity would be cut down significantly if all the above equipment could be replaced by a static optical setup consisting of LEDs and filters powered by simple electronic devices. Several previous reports have described the use of LEDs for microscopy [9]–[11], and some commercial sources are becoming available. In this paper we present simple procedures to couple LED based illumination sources to a standard widefield Olympus microscope and focus on one imaging application that employs blue and infrared LEDs.

## Results

### Making a Basic Illuminator

LEDs can be effectively used as illuminators in a standard microscope. The simplest case is the one where only a single illuminating wavelength is required. For this purpose, one can match and even exceed the illumination provided by Xenon lamps through either of two simple coupling methods to be discussed later. The main concerns here are adequate cooling, and importantly, use of a DC regulated power supply.

### Mounting, power supply and circuitry

**Figure 1:**
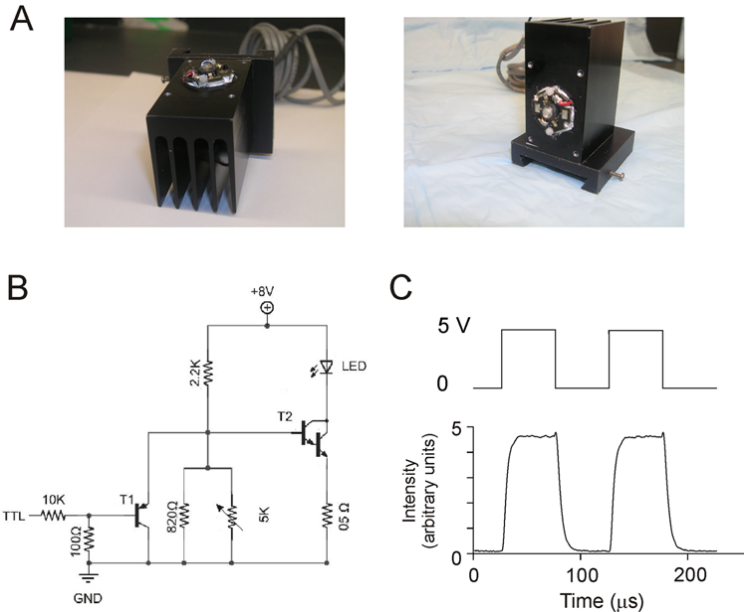
A high intensity Luxeon V LED and driving circuitry. (A) A high intensity 5 W Luxeon V blue LED is shown mounted on a standard fin type heatsink with thermal grease and an electrical insulator in between. There are no moving parts used, reducing susceptibility to mechanical damage. (B) A simple electronic circuit that can be used to drive a single blue LED is shown. Its output intensity is adjusted by a potentiometer; the light can be turned on and off by a low current TTL input. (C) An LED driven by a high square wave input follows the signal closely. The speed of a typical LED array is clearly illustrated by its response to a voltage step. The driver circuit was that shown in B.

Cooling can be achieved by a large surface area fin type heat sink as shown in Figure 1A, commonly used for CPUs in computers, with additional use of a fan if the LED is to be used in a small confined space. The LED and the heat sink should be thermally connected using thermal grease available from any electronic supply house and a thermally conductive electrical insulator (e.g. mica). Although LEDs generate far less heat than arc lamps (normalized to the light output), temperature-dependence of emission necessitates careful experimental design when LED power is turned on and off as a way of shuttering image acquisition.

The power supply must be chosen with care to allow stable illumination, as well as rapid modulation of intensity (even if it is simply turning light on and off). Illustrated in Figure 1B is a sample voltage driver circuit for controlling a high intensity blue Luxeon LED with a simple low current 5 V TTL input as the switching signal. This signal can be obtained from an IO board, the serial port, or the parallel port on a computer. The light intensity is controlled by the potentiometer. Care must be taken to stay within the safe maximum driving current (usually specified by the manufacturer). Unfortunately, LEDs of the same model can vary substantially (∼1 V) in their forward voltage drop (Vf) due to differences in manufacturing and inhomogeneities in the raw building materials. This translates into differences in the current that different LEDs from the same “batch” pass at a particular holding voltage. Therefore, to avoid damaging the LEDs, when using a voltage source, one should determine in advance the holding voltage to current relationship for any given LED unit. Then, simply adjust the potentiometer or the holding voltage of the controlling circuit accordingly so that the maximum recommended current is not exceeded (Figure 1B). Alternatively, to maximize the LEDs performance, inexpensive current regulated LED power supplies can be used (for example, 3021/3023 “BuckPuck” driver, wiring diagrams and datasheets can be found at: http://www.leddynamics.com/LuxDrive/datasheets/3021-BuckPuck.pdf, or the MAX16818 current driver from http://www.maxim-ic.com/.

The responses of an LED driven by a minimal electronic circuit consisting of a single transistor and 0.5Ω resistor are shown in Figure 1C. We recorded the light intensity using a photomultiplier tube (R3896+C270 DAP socket, Hamamatsu Photonics, K.K., Hamamatsu City, Japan) and an IO DAQ board (PCI6110E, National Instruments Corporation, Austin, TX). Not surprisingly, LEDs driven at high rates (10 KHz) can follow these changes well. The on/off kinetics appears to be dominated by time constants of electronic components from the driving circuit of choice – it is around 10 µs in this present example with a rather simple, suboptimal circuit.

### Coupling the light source to the microscope

The LED should be coupled to a microscope or used to illuminate the specimen directly. A few schemes for coupling of the light source are described and illustrated below. While designing coupling optics, a key consideration is the so-called Lagrangian invariant, or more precisely a related quantity called étendue. Étendue is proportional to the product of the source size and the divergence angle of the emission, and provides an estimate of the maximum amount of light that can be collected through an aperture. Proper acknowledgement of this invariant is useful in avoiding unnecessary or impossible coupling. The reader can find a thorough discussion of invariants in Born and Wolf [12], or a more introductory discussion in Fischer and Tadic-Galeb [13].

**Figure 2:**
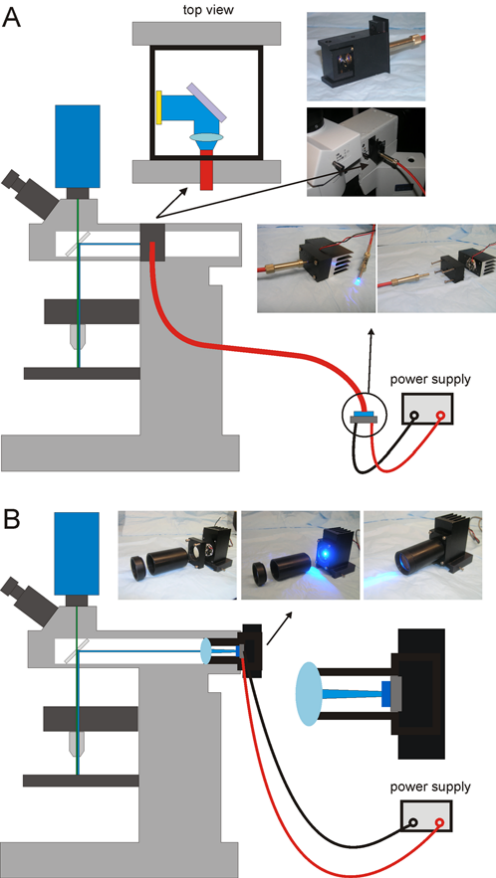
Two methods for coupling an LED based illuminator to a microscope. (A) An optical is fiber placed on the surface of an LED to direct light into a small box consisting of a focusing lens and mirror that is inserted into a side port on the microscope. (B) A homemade “lamp” consisting of an LED, heatsink, tube, and lens is coupled to the lamp housing port in the back of the microscope.

One can use a light guide, such as Edmund Optics's Liquid Light Guides (NT53-691, Edmund Optics, Inc., Barrington, NJ) to transfer the light and shine it onto a wide area or to direct the light from the LED to a desired location such as to a microscope (Figure 2A). The placement of the optical fiber or light guide in relation to the LED is important to consider. For surface emitting LEDs whose emitting surface is the same as the fiber size, one can simply place the light guide as close to the surface of the LED as possible (butt-coupling). If the fiber size is smaller, then some coupling optics can be used to match the size of the fiber to the source. But this will only help when the étendue of the fiber is greater than or equal to that of the source. A nice discussion of these issues can be found in an article by Doric and Tubic in the October 2004 issue of Laser Focus World (reprint also found in http://www.doriclenses.com/lire/39.html). For all practical purposes, the butt-coupling approach allows for the best light collection and dispenses with unnecessary and expensive optics.

Since the excitation and emission wavelengths are quite close for some fluorophores, an excitation filter should be used with the LED. In this case, the filter could be placed either directly on the LED surface, possibly sandwiched between the LED and the light guide, be mounted at the other end of the fiber, or in a traditional filter wheel if a microscope is to be used. Alternately, one might also rely on the existing filter cubes in the microscope if they match the emission wavelength of the LED arrays. The exact configuration depends on whether the light source is coupled to a microscope or used as a standalone illumination source.

Figure 2A shows a simple method to couple LED lighting sources to an Olympus BX51WI fixed stage microscope (Olympus America, Inc., Center Valley, PA). A high intensity blue LED from Lumileds (Luxeon LXHL-LB5C peak emission at 470 nm) was coupled to a 1 mm optical fiber (BFH48-1000 Thorlabs, Inc., Newton, NJ) Using the fiber optics, light from the LED is sent to the microscope into one of the slots meant for the neutral density filters as shown in the figure. There, a mirror is used to align the light onto the optical path that is normally used by a xenon lamp attached to the the microscope via a light guide (Polychrome V from Till Photonics). If an excitation filter was not used on the LED, one can be placed in the microscope at this location or in the filter wheel.

In the alternate coupling method, shown in Figure 2B, the same LED and driving circuitry are attached to a tube and lens, and coupled to the microscope through the rear lamp housing port. The tube length and the lens should be chosen specifically so that the principles of Köhler illumination are obeyed. This will ensure homogeneous illumination of the specimen. In practice, this involves mounting the tube such that it can be translated smoothly to allow focusing of the LED and following procedures similar to those used for arc lamps. A less effective, but simpler way of reducing inhomogeneity involves the usage of a diffuser placed between the LED and the collection lens. With this combination, the tube position can be adjusted to achieve maximum brightness. Any residual inhomogeneity in illumination can be corrected (in principle) by applying flat field correction digitally (methods can be found in standard optical microscopy or image processing text books). In our laboratories, we have used lens (LA1805-A), tube (SM1L30) and diffuser (DG10-600) from Thorlabs.

If the LED is to be used as a standalone illumination source (rather than through a commercial microscope's optical relay), one simply needs to consider whether to have focusing or expanding lenses attached at the end of the light guide and whether to include an excitation filter in the light path.

### Two Wavelength Illuminator

When more than one excitation wavelength is desired, independent electronic circuits can be constructed to drive multiple LED arrays similar to that shown in Figure 1B, along with a small optical relay to couple two different LEDs onto a single optical fiber bundle. While it is possible to adjust the intensity of the LEDs from the software using pulse width modulation, for most experiments requiring simply an on or off state for each LED, the intensity can be much more easily regulated in this circuit with a potentiometer.

**Figure 3:**
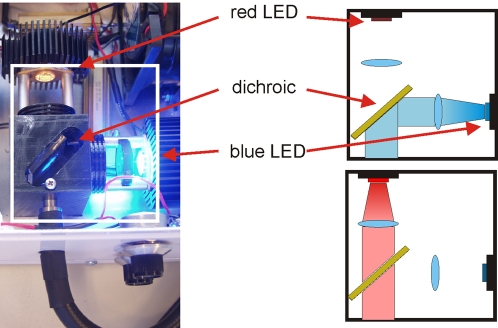
A standalone two wavelength illuminator. Two different LEDs, with independent electronic driving circuitry similar to that shown in Figure 1 are used. A dichroic mirror passes light from one LED and reflects light from the other onto the end of the fiber light guide. Any necessary excitation filters can be placed on the LEDs.

The optical setup shown in Figure 3 has two LEDs with appropriate excitation filters mounted (D480/20, Chroma Technology Corporation, Rockingham, VT). The output light is focused and reflected off a dichroic mirror that allows passage of one wavelength (780 nm), while reflecting the other (470 nm). Both light sources are thus aligned onto the edge of the light guide or optical fiber used.

### Comparison of arc lamps with LEDs

**Figure 4:**
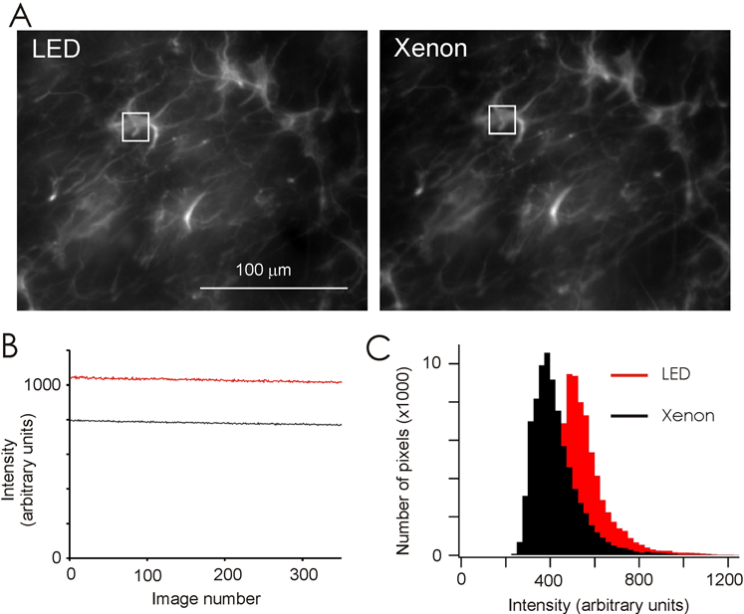
Comparison of an LED based illuminator and Xenon arc lamp. (A) Images of hippocampal astrocytes in cell culture labeled with antibodies against the astrocytic marker GFAP are shown for illumination with Xenon lamp and with LED. (B) Time traces of fluorescence intensity of the same region of the field (shown by white square) in the two conditions. (C) Distribution of pixel intensities of the images.

We compared fluorescent images of fixed tissue sections labeled with antibodies against the astrocytic marker GFAP, obtained sequentially using an LED driven at 700 mA and a xenon-arc lamp (TILL Polychrome, TILL Photonics, LLC., Pleasanton, CA). The LED was coupled to the microscope by simply placing one end of an optical fiber on the LED chip. The dichroic, objective lens and emission filter were the same for both light sources, as was the exposure time of 25 ms. The images and intensity plots in Figure 4 illustrate that, for matching imaging conditions, the LED signal was 30% brighter and as uniformly focused on the sample as the xenon-arc signal. Improvements in light coupling, transmission, and LED technology will likely lead to even brighter LED based light sources.

A similar comparison with a 100 W mercury lamp revealed that the output power of an LED array at the focal plane of the objective was approximately ten times less than that of a mercury lamp. Thus, in terms of available output, top end LEDs currently cover a range of intensities between the xenon and mercury lamps. Their output power is however sufficient for most biological applications, including imaging of weakly fluorescent probes like synaptopHluorins (see below).

### Custom-built widefield microscope for in vivo imaging

In this final example, we present a sample application in which real-time fluorescence imaging can be done in an intact neural system using LED illumination. The preparation is the mouse olfactory bulb [14]. Each glomerulus in the adult olfactory bulb receives input from a single type of odorant receptor (∼1300 types in mice). Within the glomerulus, tens of thousands of sensory axons make synapses on principal cells and interneurons. Glomeruli are located a variable distance below the surface of the bulb, ranging from 10 µm to a 100 µm; each glomerulus is about 80 µm in diameter in the mouse. Odorants bind to receptors in the nose and activate sensory neurons, which then release neurotransmitter in the glomerulus. Thus each glomerulus on the surface of the bulb has a specific chemical response spectrum, derived from the associated odor receptor.

Two optical signals are used to measure responses in glomeruli - intrinsic optical signals, derived from changes in near infrared light scattering [15]–[17], and fluorescence signals from either calcium indicators [18] or the presynaptic probe synaptopHluorin (SpH) [14], [19]. Here we show measurements of intrinsic optical signals and synaptopHluorin signals.

**Figure 5:**
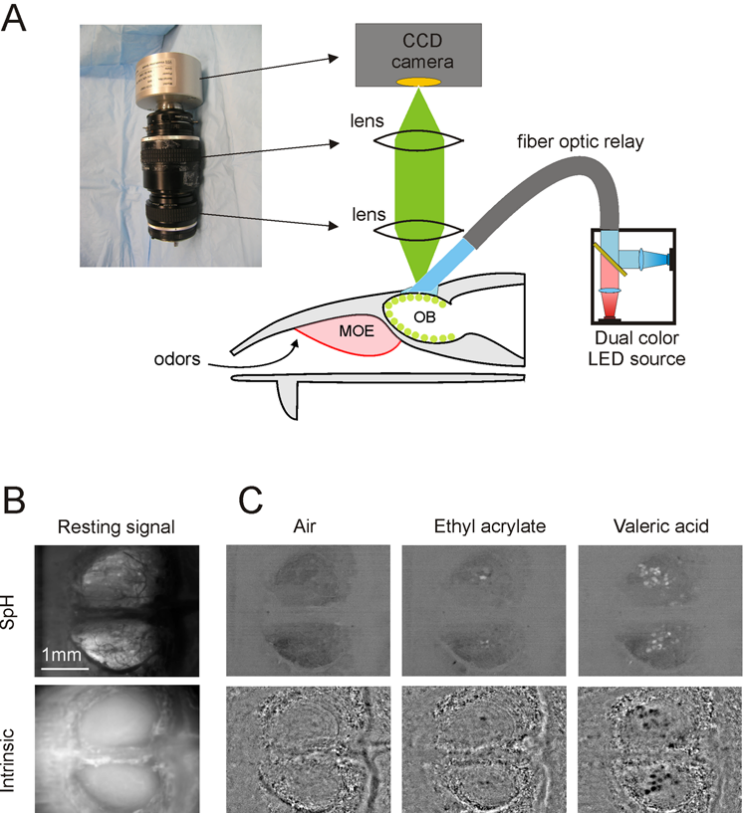
A custom built widefield microscope. (A) As an alternative to a standard commercial microscope, we used a custom built setup as shown above for our *in vivo* widefield experiments. A CCD camera is coupled to two lenses along with optional extension tubes to adjust the magnification. The ordering of parts is as follows: CCD camera, an F to C mount adapter with an emission filter placed inside or on top of the coupler, a 62 mm, 105 mm FL lens, a 62 mm to 52 mm step down ring, a 52 mm to 52 mm coupling ring, and a 52 mm, 50 mm FL lens. (B) Shown here are sample individual frames of the dorsal surface of the olfactory bulb, under white light, revealing the blood vessel architecture, as well as resting synaptopHluorin and intrinsic optical signals from the same animal respectively under blue and far red illumination. (C) The changes in spH and intrinsic signals upon presentation of fresh air or two odorants. Images show fractional changes (ΔF/F) with increases shown by brighter pixels and decreases by darker pixels.

To image olfactory bulbs, we coupled two lenses (Nikon Telephoto 105 mm f/1.8 AIS Manual Focus Lens and Nikon Normal 50 mm f/1.2 AIS Manual Focus Lens, Nikon Corporation, Melville, NY) using a 62 mm to 52 mm stepdown ring and a 52 mm to 52 mm coupling ring. This system of lenses was attached to a CCD camera (VDS Vosskuhler CCD-1300F, VDS Vosskuhler GmbH, Osnabruck, Germany) using an F to C coupler. An optional set of extension tubes can be used between the F to C coupler and the 62 mm lens to adjust magnification. This optical relay was chosen to facilitate imaging over a large area and depth of field, minimize light loss, and to provide flexibility with different lenses. A standard macro lens could be used for the same purpose, but is more expensive and less flexible than the lenses we use. To allow fast switching between two wavelengths, we assembled the box depicted in Figure 3 that would hold LEDs of two wavelengths with an appropriate dichroic mirror to direct light to a quartz fiber optic light guide (NT38-956 Edmund Optics). To collect signals in the wavelength range from green to near infrared, we used a longpass filter (HQ510LP, Chroma Technology). Photographs and schematics of the device are shown in Figure 5A.

Imaging was performed as in Meister and Bonhoeffer [16]. Mice were anesthetized with a ketamine/xylazine mixture. A craniotomy was performed in which the skull over the olfactory bulb was removed. A 1% agarose solution and glass coverslip were placed on top to support the bulb and prevent damage. Either blue or red light from an LED array was directed over the olfactory bulb using flexible fiber optic bundles (Figure 5A). Images of green fluorescence and red backscattered light were acquired using a CCD camera. Figure 5B illustrates the baseline signals obtained under infrared and blue lights, along with corresponding intrinsic and spH responses evoked by odor applications. Odors were delivered using a custom-built olfactometer [16]. The blue and infrared LED arrays were toggled on and off sequentially, such that both intrinsic and fluorescence signals could be obtained in the same individual trial. Intrinsic signals were passed through a spatial filter kernel as described in Meister and Bonhoffer [16] to isolate the high spatial frequencies corresponding to individual glomeruli odor triggered responses. SpH fluorescence was directly analyzed without further processing. As illustrated in Figure 5B, odor-evoked changes in both fluorescence and intrinsic optical signals can be easily detected using LEDs as an illumination source. These experiments demonstrate the feasibility of quantitative real-time imaging in a relatively complex biological preparation.

## Discussion

As LED manufacturing processes become more advanced, cheaper and brighter LEDs will become available in the near future. The methods described here should allow investigators to exploit the advances in LEDs and construct inexpensive and easy-to-use light sources for optical microscopy.

## Materials and Methods

We provide a list of parts used for the assembly of the illuminator here, and describe the actual logic and process of assembly in the Results section.

For microscope attenuation filter slot coupling, the following parts were used:

1. Chroma Technology HQ510LP; long pass emission filter
2. Edmund Optics NT52-539; filter dichroic mag 12.5 MM D subtractive magenta 12.5 mm diameter
3. Thorlabs BFH48-1000; multimode Fiber, 0.48 NA, High OH, 1000 µm Core
4. Thorlabs ME05-P01; 12.7 mm Dia. Round Protected Silver Mirror
5. BK precision model 1626A Power Supply DC regulated power supply - 3Amp, 30 V,
6. Lumileds LXHL-LB5C; blue LEDs - lambertian - centered at 470 nm
7. Lumileds ATH 652453B1200; heat sink

For microscope lamp housing coupling, the following parts were used:

1. Chroma Technology; HQ510LP; long pass emission filter
2. Edmund Optics NT52-539; filter dichroic mag 12.5 MM D subtractive magenta 12.5 mm diameter
3. Thorlabs SM1L30; SM1 Lens Tube, 3″ Long,
4. Thorlabs LA1805-A; BK7 A Coated Plano-Convex Lens, DIA = 25.40 mm, f = 30.00 mm
5. BK precision model 1626A Power Supply DC regulated power supply - 3Amp, 30 V,
6. Lumileds LXHL-LB5C; blue LEDs - lambertian - centered at 470 nm
7. Lumileds ATH 652453B1200; heat sink

For the custom widefield imaging setup, the following parts were used:

1. BK precision model 1626A Power Supply DC regulated power supply - 3Amp, 30 V,
2. Lumileds LXHL-LB5C; blue LEDs - lambertian - centered at 470 nm
3. Lumileds ATH 652453B1200; heat sink
4. Roithner Laser Technik LED 780-66-60 – far red LEDs centered at 780 nm
5. Roithner Laser Technik, Heat sink 47×20 TO-66 Aluminum black anodized
6. Roithner Laser Technik, lens for LED, LED-OPTIC-12 aspheric glass, 12 mm FL
7. Edmund Optics cold mirror (dichroic) H43961
8. McMaster Carr, Flexible Arm Magnetic Base with fine adjustment (20755A65) to hold the LEDs
9. VDS Vosskuhler CCD 1300-F QE; CCD camera
10. Dynamic Engineering, http://www.dyneng.com, Custom SCSI Cable VDS-DSub 100 pin for connecting the CCD camera to an acquisition board (National Instruments PCI1422 LVDS)
11. Nikon Normal 50 mm f/1.4 AIS Manual Focus Lens
12. Nikon Telephoto 105 mm f/1.8 AIS Manual Focus Lens
13. Nikon F to-C Lens Mounting Adapter
14. Tiffen 62 mm-52 mm Step-Down Ring
15. Rokunar 52 mm male to male ring macro coupler
16. Newport Rod 40 (parts 16-21 are to hold and translate the CCD camera)
17. Newport Rod Platform 300-P
18. Newport Z stage M-461-Z-M
19. Newport SM-13 actuator
20. Thorlabs RP01 - Rotation Platform, Lockable, Thorlabs
21. Linos STOP Ring 38 - 024948

## References

[1] Deisseroth K, Feng G, Majewska AK, Miesenbock G, Ting A (2006). Next-generation optical technologies for illuminating genetically targeted brain circuits,. J. Neurosci..

[2] Giepmans BN, Adams SR, Ellisman MH, Tsien RY (2006). The fluorescent toolbox for assessing protein location and function.. Science.

[3] Tsien RY (2003). Imagining imaging's future (2003) Nat.. Rev. Mol. Cell. Bio. (Suppl).

[4] Lichtman JW, Conchello JA (2005). Fluorescence microscopy,. Nat. Methods.

[5] Holman B (2007). LED light source: major advance in fluorescent microscopy.. Biomed. Instrum. Technol..

[6] Round HJ (1907). A note on carborundum,. Electrical World.

[7] Zheludev N (2007). The life and times of the LED – a 100-year history.. Nat. Photonics.

[8] Salzberg BM, Kosterin PV, Muschol M, Obaid AL, Rumyantsev SL (2005). An ultra-stable non-coherent light source for optical measurements in neuroscience and cell physiology,. J.Neurosci. Methods..

[9] Herman P, Maliwal BP, Lin HJ, Lakowicz JR (2001). Frequency-domain fluorescence microscopy with the LED as a light source.. J Microsc..

[10] Martin G, Agostini HT, Hansen LL (2005). Light emitting diode microscope illumination for green fluorescent protein or fluorescein isothiocyanate epifluorescence.. Biotechniques.

[11] Bormuth V, Howard J, Schaffer E (2007). LED illumination for video-enhanced DIC imaging of single microtubules.. J Microsc..

[12] Born M, Wolf E (1999). Principles of Optics. (7th edition).

[13] Fischer RF, Tadic-Galeb B (2000). Optical System Design.

[14] Bozza T, McGann JP, Mombaerts P, Wachowiak M (2004). In vivo imaging of neuronal activity by targeted expression of a genetically encoded probe in the mouse,. Neuron.

[15] Rubin BD, Katz LC (1999). Optical imaging of odorant representations in the mammalian olfactory bulb,. Neuron.

[16] Meister M, Bonhoeffer T (2001). Tuning and topography in an odor map on the rat olfactory bulb,. J. Neurosci..

[17] Grinvald A, Lieke E, Frostig RD, Gilbert CD, Wiesel TN (1986). Functional architecture of cortex revealed by optical imaging of intrinsic Signals,. Nature.

[18] Wachowiak M, Cohen LB (2001). Representation of odorants by receptor neuron input to the mouse olfactory bulb.. Neuron.

[19] Miesenbock G, De Angelis DA, Rothman JE (1998). Visualizing secretion and synaptic transmission with pH-sensitive green fluorescent proteins,. Nature.

